# Sensory Assessment of Hay Samples: Abnormal Odor Predicts Increased Dust Levels and Impurities Suggest Microbiological Contamination

**DOI:** 10.3390/ani15182688

**Published:** 2025-09-14

**Authors:** Virginie Marie Angèle Bouverat, Nicolas Pradervand, Brigitta Annette Wichert, Eloïse Greim, Gaudenz Jürg Dolf, Vinzenz Gerber

**Affiliations:** 1 Swiss Institute of Equine Medicine (ISME), Department of Clinical Veterinary Medicine, Vetsuisse-Faculty, University of Bern, 3012 Bern, Switzerland; eloise.greim@unibe.ch (E.G.); gaudenz.dolf@bluewin.ch (G.J.D.); 2 Feed Biology Laboratory, Division of Methods Development and Analytics, Agroscope, 1725 Posieux, Switzerland; nicolas.pradervand@agroscope.admin.ch; 3 Institute of Animal Nutrition and Dietetics, Vetsuisse Faculty, University of Zurich, 8057 Zurich, Switzerland; bwichert@nutrivet.uzh.ch

**Keywords:** forage hygiene, sensory evaluation, particulate matter, microbiology, respiratory health

## Abstract

Hay quality is a key factor in equine respiratory health, with microbiological contaminants in inhaled organic dust posing significant risks. Sensory assessment has been used to evaluate hay hygiene, but its value to identify deficiencies remains unclear. This study aimed to explore the potential of sensory assessment to predict both particulate matter (PM) dust concentrations and microbiological contamination. Fifty hay samples were collected from horse owners and evaluated using a structured sensory examination, microbiological analyses, and dust concentration measurements obtained with the Hay-Shaker device and a DustTrak DRX 8534. Sensory examination rated only 28% of samples as adequate, with 52% showing minor and 20% major deficiencies. Microbiological analysis found that 46% of samples met acceptable standards. Regression analysis showed that abnormal musty odor was the strongest predictor of increased dust concentrations, including the respirable fraction (PM4, <4 µm), while visible impurities were associated with microbial contamination. These findings suggest that sensory attributes such as odor and impurity are valuable indicators of hay hygiene. Structured protocols for sensory examination may offer a simple and cost-effective tool for assessing hay quality in equine environments.

## Simple Summary

Hay is a major part of a horse’s diet, but if it contains too much dust or is contaminated with harmful microorganisms like mold or bacteria, it can cause serious breathing problems. Horse owners and caretakers often check hay quality using sight, touch and smell, but it is unclear whether this simple method can detect hay that might be harmful. In this study, researchers collected 50 hay samples from horse owners and examined them using both sensory examinations, as well as specific methods to measure dust levels and check for potentially harmful microorganisms. The results showed that hay with an abnormal smell was more likely to produce high levels of dust, and hay with visible dirt or debris was more likely to contain potentially harmful bacteria or fungi. This research highlights the value of basic sensory assessments as a rapid and low-cost means to monitor hay hygiene and protect horses’ respiratory health.

## 1. Introduction

Hay is a major component of the equine diet, and its quality can significantly impact equine health, particularly of the respiratory system [1,2]. Poor-quality hay, particularly if contaminated with harmful microorganisms or debris, has been associated with airway inflammation and the development of equine asthma, a common respiratory condition among horses, affecting up to 50% in certain regions [3,4].

Hay quality is influenced by both its composition and potential contamination. It is important to differentiate between field flora—microorganisms naturally present on plants during growth—and spoilage flora, which proliferates under poor harvesting or storage conditions and may pose greater risks to respiratory health [5,6]. Contaminants can be classified into biological (toxic plants, dust, insects, bacteria, toxins), chemical (fertilizers, heavy metals, etc.), and physical (sand, glass, etc.) [7]. Among these, biological contaminants are of particular concern due to their presence in respirable organic dust. Inhalation of such organic dust—including mold spores, bacterial endotoxins present in fine particulate matter (PM)—has been widely recognized as a major contributor to lower airway inflammation and asthma exacerbation in horses [6,8,9]. The impact of these contaminants on respiratory health depends largely on the size of the airborne particles, which determines how deeply they can penetrate into the respiratory tract. These PM fractions describe the thoracic fraction (PM10, <10 μm) reaching the trachea and bronchi, the respirable fraction (PM4, <4 μm) reaching the bronchioles and the alveolar fractions (PM2.5 and PM1, <2.5 and <1 μm, respectively) that penetrate to the alveoli.

Experimental studies using dusty or moldy hay or nebulized hay dust suspensions consistently replicate the clinical and inflammatory features of equine asthma, especially in susceptible individuals [10,11,12]. Particles smaller than 4 μm (PM4) are especially hazardous, as they can reach the distal airways and trigger inflammatory responses [11,13,14,15,16]. A previous study found a positive association between visually apparent dust and elevated respirable particle levels, with dusty or moldy hay releasing significantly more PM compared to visually clean hay [17]. Mold spores and bacterial endotoxins in hay dust are widely recognized as key etiological components of this syndrome [18,19,20,21,22,23]. Among mold species, *Aspergillus fumigatus* is particularly relevant, as this mold produces spores capable of triggering severe asthma symptoms [14,24].

Two primary methodologies are employed to assess hay quality: sensory examination (also called organoleptic) and microbiological analysis. Sensory assessments, such as the protocol for sensory examination of roughage [25], offer practical advantages due to ease and speed, allowing early identification of potentially problematic forage. However, sensory examinations are inherently subjective and may fail to detect subtle microbiological contamination or quantify dust exposure accurately. Consequently, microbiological analyses, referencing established standards (Verband Deutscher Landwirtschaftlicher Untersuchungs und Forschungsanstalten, VDLUFA) [26], represent the gold standard to quantitatively evaluate bacterial, fungal, and yeast contents in hay samples.

The relationship between sensory evaluation and objective measures of hay quality remains poorly defined. Previous studies examining agreement between sensory and microbiological assessments yielded mixed results. Intemann et al. [14] found moderate correlations between sensory-detected abnormal odor and microbiological contamination levels, whereas Stickdorn et al. [27] reported weaker associations overall but noted trends relating sensory deficiencies to mold and yeast contamination. Importantly, no studies have directly examined whether sensory evaluation can predict dust release, despite its relevance for equine respiratory health. Thus, the practical value of sensory assessment as a predictive tool for hay safety remains uncertain.

This exploratory study aims to investigate the value of specific sensory attributes assessed using the adapted Kamphues protocol [25] to predict objective measures of hay quality, specifically dust concentrations and microbiological contamination. We hypothesized that certain sensory attributes would be positively associated with higher dust concentrations and greater microbiological contamination. By exploring sensory examination as a predictive tool, this research seeks to enhance the practical applicability of simple on-site evaluations for routine hay screening, thereby contributing to improved equine respiratory health management and prevention strategies.

## 2. Materials and Methods

### 2.1. Study Design and Sample Collection

This exploratory study employed a cross-sectional design to investigate the associations of sensory attributes with microbiological contamination and with dust concentrations in hay samples. Data on these hay samples, particularly regarding dust concentrations, were collected in a previous experimental study conducted at the Swiss Institute of Equine Medicine in Avenches, Switzerland, from April to October 2023, in which measurements obtained with a Hay-Shaker were compared to corresponding measurements in the horses’ breathing zone [28]. Fifty hay samples, each weighing approximately 5 kg, were collected by horse owners from recently opened hay bales. The hay samples consisted of untreated dry hay. Each hay sample was manually homogenized by gently mixing it in a large 110 L garbage bag. The homogenized material was then subdivided into two 1 kg sub-samples. During this process, portions were taken from the top, middle, and bottom of the bag to account for dust settling. Each sub-sample was placed in a separate 110 L garbage bag for subsequent sensory evaluation and dust analysis, with gentle mixing performed again in the smaller bags to ensure uniformity. Samples were weighed using a digital luggage scale (INTERTRONIC Digital Pèse-bagages, InterDiscount, Jegenstorf, Switzerland). Additionally, a 200 g sub-sample was prepared for microbiological analysis, weighted with a precision scale (SOEHNLE 65105 1 Style, Soehnle Industrial Solutions GmbH, Backnang, Germany) and sent to the Posieux Agroscope laboratory on the same day of collection.

### 2.2. Data Collection

#### 2.2.1. Sensory Examination

**Figure 1:**
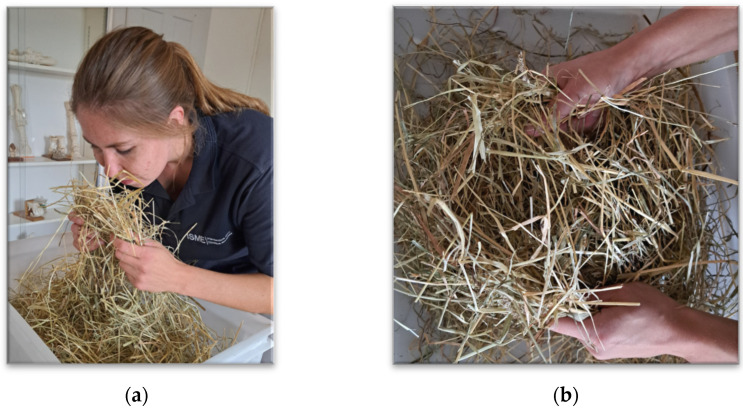
Illustration of sensory examination including odor, texture, color and impurities. (**a**) The examiner assessed the odor of the hay; (**b**) Hay color, and the presence of impurities were assessed visually, and haptic assessment (sense of touch) was used to evaluate texture.

The sensory examination was conducted according to the Kamphues protocol [25] (as described in Table 1), assessing four specific criteria: odor, texture, color, and visible impurities (Figure 1). Two trained examiners performed independent assessments blinded to the results of the other analyses. Scores were assigned for each attribute according to standardized criteria, with deductions ranging from 0 to −5 (texture and color), and from 0 to −10 (odor and impurities).

#### 2.2.2. Measurement of Dust Generated with the HS

A detailed description of this Hay-Shaker and its measurements are given in Bouverat et al. [28]. Dust concentrations were measured using the Hay-Shaker device in combination with the DustTrak DRX 8534 (TSI Incorporated, Shoreview, MN, USA) to generate dust from hay under controlled conditions independent of horse-related factors. The equipment provides real-time data on PM fractions, including PM1, PM2.5, PM4, PM10, representing particle size in μm, and total PM (PMT) in mg/m^3^. Each measurement was performed over a period of 10 min, with one data point recorded every second, resulting in a total of 600 values for each PM fraction per horse. Measurements were calibrated with ISO 12103-1 A1 [29] test dust and using airflow zero calibrations before and after each experiment, and the testing environment was controlled for consistent data collection, as described in detail by Bouverat et al. [28].

#### 2.2.3. Microbiological Analyses

**Sample preparation and platings.**

The 50 hay samples were analyzed for the microbiological quality according to the VDLUFA method 28.1.1, 28.1.2, 28.1.3 and 28.1.4 [26]. Hay samples were manually cut down into approximatively <5 cm pieces with disinfected scissors. Twenty grams of the cut-down sample (including dust) were added to 380 mL of buffered peptone water (Millipore, Billerica, MA, USA, 1.07228) into a filtered stomacher bag, let soak for 5 min and homogenized with a stomacher for 5 min. The suspension was then decimally diluted. 0.1 mL of each dilution were pipetted onto two Petri dishes filled with appropriate culture media and evenly spread. The culture medium for aerobic mesophilic bacteria was Tryptose agar (Sigma-Aldrich, St. Louis, MO, USA, T2313) supplemented with 30 µM 2,3,5-Triphenyltetrazolium chloride (Sigma-Aldrich, St. Louis, MO, USA, 17770). The first culture media for yeasts and molds was Dichlorane Glycerol Agar (DG18 Agar, Oxoid, Basingstoke, UK, CM0729) supplemented with 120 µM oxytetracycline hydrochloride (Sigma-Aldrich, St. Louis, MO, USA, O5875). The second culture media for yeasts and molds was Rose-Bengal Agar (Oxoid, Basingstoke, UK, CM0549) with Tergitol (0.01% final) and 120 µM oxytetracycline hydrochloride (Sigma-Aldrich, St. Louis, MO, USA, O5875). The inoculated Perti dishes were then incubated upside-down according to the VDLUFA method (30 °C, 2 days for bacteria and 25 °C, 3 days for yeasts and molds, respectively).

**Counting and classification of micro-organisms.**

The colony-forming units (CFU) of aerobic mesophilic bacteria (AMB), molds and yeasts were each counted on their respective media (with regard to the dilutions they originated from). Petri dishes with CFU numbers ranging between 20 and 200 CFUs were considered. The obtained concentrations were given in CFU per gram. Then, each CFU was classified into 7 categories of micro-organisms according to their ecology and hazards: Group 1 represents the product-typical bacteria (such as members of the genera *Pantoea*, *Enterobacter*, *Pseudomonas* and *Enterobacteriaceae* to name a few). Group 2 includes the spoilage-indicating bacteria (including members of the genera *Bacillus*, *Staphylococcus*, *Micrococcus*). Group 3 concerns the spoilage-indicating *Streptomycetes* spp. Group 4 includes the product-typical fungi, living “on the field” and represented, among others, by the *Dematicaeae* and the genera *Verticillium*, *Acremonium*, *Aureobasidium* and *Fusarium*). Group 5 encompasses the spoilage-indicating molds, like the genera *Penicillium*, *Aspergillus*, *Wallemia* and others. Group 6 is formed by the spoilage-indicating *Mucorales* (like *Mucor* spp., *Rhizopus* spp. or *Absidia* spp.). Finally, Group 7 is made of all species of yeasts, which are also considered indicators of spoilage.

**Assessment of the overall microbiological quality of hay.**

The calculated concentrations of each of the 7 groups in a sample were compared to the orientation values for hay, as established by the VDLUFA method 28.1.4. The given orientations values for hay are shown in Table 2 (version 2024). If the concentrations of one (or more) of the 7 groups found in a sample were equal or below the concentrations given in the orientation values, the hay was considered “normal” (grade 1) in terms of microbiological quality. If the orientation values were exceeded up to 5 times by the concentrations found in the sample (in any of the 7 groups), the hay received a “slightly reduced” microbiological quality (grade 2). If the orientation values were exceeded up to 10 times by the concentrations found in the sample (in any of the 7 groups), the hay received a “distinctly reduced” microbiological quality (grade 3). If the orientations values were exceeded more than 10 times, then the microbiological quality was assessed as “significantly altered” (grade 4, i.e., not acceptable for feeding).

### 2.3. Data Processing

For statistical analyses we considered the mean PM levels (PM1, PM2.5, PM4, PM10, PMT described above in Section 2.2.2. and in [28] as the first continuous outcome variables. The other outcome variable, microbiological quality, entered the analyses as a categorical characteristic (grades 1–4) as outlined above in Section 2.2.3. Three types of summary ratings were generated from the sensory examination to assess hay quality. Rating 1 (Adapted from the protocol of Kamphues et al. 2024 [25]) reflected the overall hay quality based on the total score, Rating 2 considered the individual scores for odor, texture, color, and impurities, and Rating 3 applied a binary classification of the same four criteria. The specific scoring systems and categories for each rating are summarized in Table 3.

### 2.4. Statistical Analyses

Statistical analyses were performed using R version 4.4.3 (R Core Team 2025; R Foundation for Statistical Computing, Vienna, Austria). For data import and export the package rio was used [30]. Descriptive statistics were computed for sensory scores (categorical) and microbiological scores (MBS, categorical), as presented in this paper. Descriptive statistics for all PM variables (continuous) are presented in Table 1 of Bouverat et al. [28].

Shapiro–Wilk tests were performed to test normality. PM measurements were square root-transformed to stabilize variance before regression analysis. Ordered logistic regressions were calculated using the package MASS [31]. To check the parallel line assumption, the package brant was used [32]. Models were compared using the package performance [33]. Parameters were extracted from the models either by hand or by using the package parameters [34]. For plotting the packages ggplot2 [35] and patchwork [36] were used. Model selection was based exclusively on the Bayesian Information Criterion (BIC). The significance level for all statistical tests was 0.05.

Agreement of sensory scores between the two examiners was assessed by Kendall’s tau-b correlation and Wilcoxon signed-rank test.

## 3. Results

### 3.1. Descriptive Statistics

#### 3.1.1. Sensory Examination

Out of 50 evaluated hay samples, according to Rating 1, 28% (*n* = 14) were classified as adequate, 52% (*n* = 26) exhibited minor deficiencies, 16% (*n* = 8) had major deficiencies, and 4% (*n* = 2) demonstrated massive deficiencies.

For Rating 2, the vast majority of hay samples (98%, 49 out of 50) scored within the acceptable range for each sensory criterion: odor (0 to −5), texture (0 to −2), color (0 to −2), and impurities (0 to −5). Only 2% (1 sample) showed deficiencies with more negative scores in all categories: odor (−5.5 to −10), texture (−2.5 to −5), color (−2.5 to −5), and impurities (−5.5 to −20).

For Rating 3, which classifies sensory criteria as acceptable (0) or problematic (1), the proportion of hay samples rated problematic was odor 52% (26/50), texture 62% (31/50), color 62% (31/50), and impurities 80% (40/50). The remaining samples were classified as acceptable, corresponding to 48%, 38%, 38%, and 20%, respectively.

#### 3.1.2. Microbiological Analysis

The microbiological analyses indicated substantial variability among samples. Overall, 46% (*n* = 23 grade 1) of hay samples were classified within normal microbiological quality limits. Slightly reduced quality was observed in 30% (*n* = 15 grade 2), distinctly reduced quality in 6% (*n* = 3 grade 3), and significantly altered quality in 18% (*n* = 9 grade 4) of the samples.

As shown in Table 4, hay samples with a score of 2 most frequently exceeded guideline values for field flora indicators, in particular for molds (80%) and AMB (20%), while yeasts were also commonly elevated (53%). As expected, samples with scores of 3 and 4 showed more frequent deviations in spoilage-related microorganisms. Among score 4 samples, 89% exceeded limits for spoilage-associated molds, 11% for spoilage-associated AMB, and 11% for Mucorales. These results indicate a shift from field flora exceedances at score 2 toward spoilage-related contamination at higher sensory scores.

### 3.2. Agreement of Sensory Examination Scores Between the Two Examiners

To evaluate consistency between the two examiners, Kendall’s tau-b was used, showing a strong correlation (τ = 0.842). An exact Wilcoxon signed-rank test revealed a small but significant difference between scores (*p* = 0.037), indicating a systematic divergence despite overall agreement, with one examiner tending to assign more severe scores than the other.

### 3.3. Regression Analyses: Sensory Predictors of Dust Concentrations

Rating 1, Rating 2 and Rating 3 were evaluated as predictors for dust concentration. A total of nine regression models (Supplementary Tables S1–S5) were constructed, varying in the combination of sensory predictors included. Across all PM fractions, models including binary sensory traits (Rating 3) consistently outperformed those based on the global score (Rating 1) or continuous sub-scores (Rating 2).

The best-performing model for PM1, PM2.5, PM4 and PMT, based on the BIC, included only “odor2”. From all the binary Rating 3 predictors, abnormal odor thus emerged as the most robust and consistent indicator of increased dust, explaining 30–40% of the variance (adjusted R^2^ values ranging from 0.31 to 0.40) across all PM fractions. Models including “Impurities2” yielded similar BIC values but did not offer significant improvement (*p* > 0.05) for these fractions, suggesting that impurity could play a role, though the limited number of data points may have influenced these results.

For PM10, however, impurity2 significantly improved model performance and was retained alongside odor, suggesting an additive effect. In contrast, texture and color provided limited predictive value across all PM fraction outcomes.

To illustrate, Table 5 presents both the initial full model (including all four binary variables) and the final model (odor2 only) for PM4.

**Figure 2:**
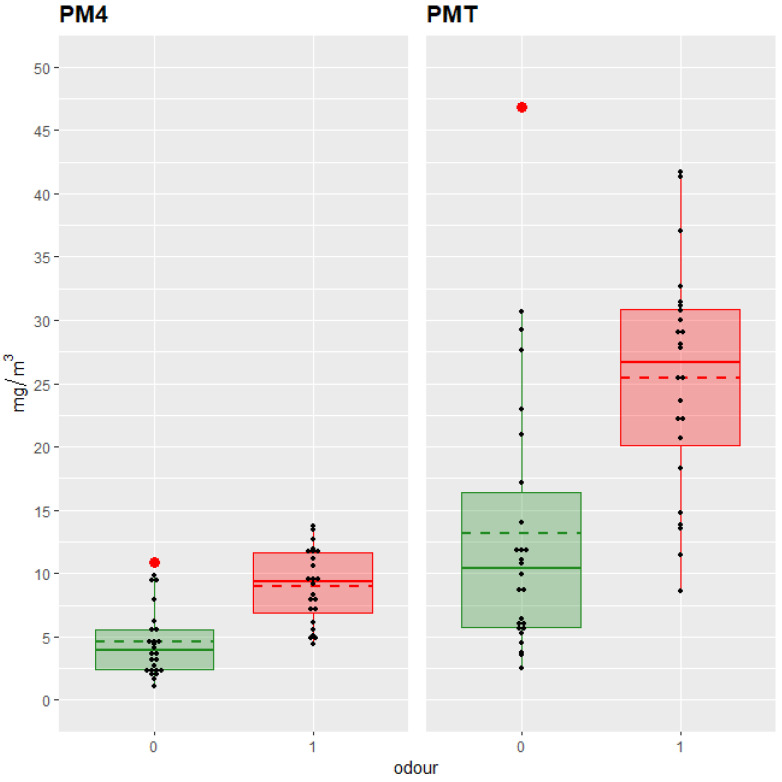
Box-and-whiskers plots of mean dust concentration (mg/m^3^) of particulate matter < 4 μm (PM4) and total particulate matter (PMT) in the Hay-Shaker by Odor (0: adequate (green boxes and lines); 1; abnormal (red boxes and lines); *n* = 50). Individual data points are shown in black, outliers are highlighted in red, and dashed lines indicate the group means.

Figure 2 illustrates this relationship for PM4 and PMT, with significantly higher dust levels observed in hay samples rated as having abnormal odor.

### 3.4. Regression Analyses: Sensory Predictors of Microbiological Quality

A total of ten regression models were evaluated to assess the predictive value of sensory assessment (Rating 1–3) for microbiological quality categories, as summarized in Supplementary Table S6. The models based on the continuous scoring of the sensory components: odor, texture, color and impurities (Rating 2) provided the best overall performance, both in terms of model fit (lower BIC) and assumption validity.

The best-fitting model (Table 6) included only impurity and showed that higher impurity levels tended to be associated with increased odds of microbiological contamination (OR = 0.541, *p* = 0.059). Despite the marginal significance, the model met the proportional odds assumption.

In contrast, models incorporating binary sensory variables (odor2, texture2, color2, impurities2) exhibited higher BIC values, indicating poorer model fit and violated the parallel regression assumption, which assumes that the relationship between the predictor variables and the outcome is the same across all levels of the outcome variable. Moreover, these binary variables were not statistically significant, suggesting that they do not contribute effectively to the prediction of microbiological contamination.

## 4. Discussion

The increasing importance of equine asthma and its links to environmental organic dust exposures underlines the need for practical and reliable tools to assess forage quality. This study addressed whether simple sensory examination could predict dust concentration and microbiological contamination in hay samples. Our findings suggest that sensory examination, particularly assessment of abnormal odor, effectively predicts dust exposure across all PM fractions, while impurities may be useful for predicting microbiological contamination.

To our knowledge, this is the first systematic study to correlate structured sensory scoring with objective dust measures across a substantial number of hay samples. Among the sensory parameters assessed, abnormal odor consistently emerged as the most robust predictor of increased dust concentrations, across all PM fractions, explaining 30–40% of the variance. This specifically included the respirable particle fraction (PM4), which has been shown to be particularly relevant as a risk factor of equine asthma [15]. This observation aligns with previous reports indicating sensory detection of musty or moldy odors typically reflects deviating germ counts [14], mold contamination [14,24] and elevated respirable dust concentrations [17]. The clinical manifestation of respiratory signs, such as coughing, has been associated with elevated mold levels in the environment, particularly in hay and stable air, with *Aspergillus* spp. identified as a relevant respiratory allergen and potential trigger of equine asthma [14,37]. While odor assessment like all sensory examination is subjective, its consistent association with PM levels suggests it holds diagnostic value for identifying hay that may pose respiratory risks.

Similarly, visible impurities, including molds, beetles, and mites, were found to be associated with microbiological contamination and showed predictive potential for PM10 concentrations, with near-significant associations for other PM fractions. This dual role supports the utility of the impurity assessment not only for identifying microbial spoilage but also for estimating dust exposure. However, the presence of such impurities does not always reflect deficiencies likely to influence dust concentration or microbiological contamination. For instance, the presence of autumn crocus (*Colchicum autumnalis*) does not necessarily indicate such issues. Similarly, blister beetles (not found in temperate climates as in Switzerland) could be directly harmful to horses. Moreover, only a limited number of hay samples with marked impurity levels were included in the study; nevertheless, these samples showed a clear trend. While our results align with Intemann et al. [14], who reported similar associations between sensory examination and microbiological analysis, it is important to note that their study included a detailed analysis of individual microbial groups (e.g., aerobic bacteria, molds and yeasts). In contrast, we focused our statistical analyses on the overall microbiological classification (grades 1 to 4) according to the VDLUFA system [26]. This classification is itself derived from the quantified exceedance of threshold values in at least one microbial category (e.g., molds, yeasts or aerobic mesophilic bacteria), thereby integrating multiple contamination indicators into a single score. While we conducted descriptive analyses of these subcategories (Table 4), we did not formally model their individual associations with sensory scores. This approach allowed us to simplify the interpretation while maintaining a quantitative and standardized assessment of microbiological quality.

The microbiological profile of hay is influenced by harvest and storage conditions [25]. While the largest proportion of hay samples was graded as adequate (grade 1), in our study, the second largest group of hay samples with a grade 2 classification (slightly reduced) were most commonly dominated by field flora components, including molds and aerobic mesophilic bacteria (AMB) which reflect the normal environmental microbiota acquired during harvest. Their overrepresentation in these samples suggests suboptimal harvest conditions, such as high humidity or mechanical damage, rather than significant spoilage. Although not strictly part of the field flora, yeasts are commonly found in these samples and can proliferate under damp or anaerobic conditions, indicating suboptimal storage. In contrast, grades 3 and 4, which combined were assigned to almost a quarter of all hay samples, were primarily characterized by spoilage-indicating organisms, including principally molds, AMB and Mucorales, reflecting advanced microbial degradation during storage under poor conditions (elevated humidity, heat or physical damage, i.e., crushing, trampling, or fragmentation). This microbial shift is accompanied by a decrease in species diversity and an increase in spoilage-indicating bacteria and molds, along with sensory changes like musty or putrid odors and visible degradation [7]. These findings illustrate how the VDLUFA classification [26] link microbial contamination levels to hay quality, providing insight into harvest- and storage-related microbial risks.

While sensory assessment is based on seemingly simple traits such as odor and visible impurities, these attributes reflect complex biological processes—such as the production of volatile organic compounds by molds or bacteria during storage, or the presence of microbial and physical contaminants. Despite its simplicity and subjectivity, sensory evaluation offers an accessible, rapid, and cost-effective screening tool for daily use in stables, especially where laboratory diagnostics are not readily available. Standardized scoring systems such as the Kamphues protocol [25] can enhance its practicality and reproducibility. Although sensory assessment cannot replace precise dust measurements or comprehensive microbiological analyses, our findings suggest it holds value as a frontline screening method to help identify hay potentially unsuitable for horses at risk of equine asthma.

However, the limited predictive value of the sensory criteria texture and color highlights the need for careful interpretation. Possibly, abnormal “musty” odor may be an early sign of spoilage, preceding changes in color or texture. Furthermore, the subjective nature of these attributes potentially accounts for their weak correlations with microbiological contamination, echoing Stickdorn et al.’s [27] findings. Although overall inter-rater correlation was good, the tests revealed significant differences in severity perception between examiners, suggesting some level of rater-dependent scoring. Including a larger number of examiners in future studies could improve the assessment of inter-rater reliability and reduce individual bias. In this context, binary scoring systems, like Rating 3, which classify traits simply as normal or abnormal, may offer a more robust and reproducible alternative by reducing the influence of subjective gradation and simplifying decision-making in field conditions. Despite statistical model validation, several additional limitations must be considered. The sample size was modest, and severely contaminated hay samples were underrepresented, which may have restricted the variability observed. Moreover, no clinical respiratory health data were collected to directly link dust or microbiological levels with equine asthma. Finally, the explanatory power of our models was modest (R^2^ = 0.31–0.40), indicating that although odor and impurity assessments showed predictive potential, their ability to explain dust and microbiological variability is limited. Taken together, these factors underline that the present results should be regarded as exploratory. Nevertheless, sensory assessment offers a rapid and practical screening tool that can help avoid feeding dusty or microbiologically inadequate hay to horses at risk for equine asthma.

Future research involving larger, more diverse datasets, a greater number of examiners for sensory examination, and direct clinical correlations is necessary to further refine sensory protocols to enhance predictive accuracy. While sensory scoring offers a rapid low-cost approach, to assess dust levels it should ideally be complemented by objective tools, such as a recently described wearable real-time particulate monitor [38]. Furthermore, the present study concentrated on hay as a major dust source and contributor to lower airway inflammation. However, it would also be valuable to investigate the relationship of sensory examination with dust levels and microbiological evaluation of straw, which is commonly used as bedding material and can significantly contribute to organic dust exposure implicated in equine asthma [39]. Notably, a survey of 46 horse farms in Switzerland, found that the hygienic standard of straw was worse than that of hay [40]. Future research should build upon these findings to optimize sensory methods for comprehensive forage and potentially bedding quality assessment.

## 5. Conclusions

The study underscores the utility of sensory examination—particularly abnormal odor and visible impurities—as an effective initial screening tool for hay quality. Incorporating such simple and cost-effective assessments into routine stable management practices could significantly contribute to improved equine respiratory health outcomes by enabling timely interventions. The sensory analysis used was essential to identify hay samples unsuitable for horse consumption due to the high proportion of moldy and even spoiled material.

## Figures and Tables

**Table 1:** Sensory examination of hay hygienic status based on the criteria odor, texture, color and impurities. The sum of the deficiencies gives a total score (sum of points deducted).

Parameter	Findings		Points Deducted
Odor	Unremarkable		0
	Musty nuances		−5
	Moldy/putrid		−10
Texture	Dry		0
	Slightly clammy		−2
	Clammy, moist		−5
Color	Product-typical		0
	Focally gray, whitish		−2
	Diffusely discolored		−5
Impurities	Visible evidence of mold, beetles, mites	None	0
		Medium-grade	−5
		High-grade	−10
	Visible evidence of poisonous plants (depending on species and amount)	None	0
		Medium-grade	−5
		High-grade	−10
Sum of points deducted			

**Table 2:** Microbiological assessment and the corresponding orientation values as colony forming units per gram (CFU/g), as established by the VDLUFA (Verband Deutscher Landwirtschaftlicher Untersuchungs- und Forschungsanstalten).

Microorganism	Group	Orientation Value (CFU/g)
Aerobic mesophilic bacteria	Field flora	30,000,000
	Spoilage-indicating	2,000,000
	Streptomycetes	150,000
Molds	Field flora	200,000
	Spoilage-indicating	100,000
	Mucorales	5000
Yeasts		150,000

**Table 3:** Overview of the three summary ratings generated from the sensory examination of hay samples. Rating 1 represents the total hay quality score, categorized into four quality levels. Rating 2 shows the individual scores assigned to each of the four criteria (odor, texture, color, impurities). Rating 3 applies a binary classification for the same four criteria, with 0 indicating acceptable and 1 indicating problematic. The table summarizes the scoring systems and categories for all three ratings.

Rating	Description	Scoring System	Categories/Range
Rating 1	Total hay quality score	Sum of all criteria (0 to −40), categorized into 4 categories	Adequate: 0 to −0.5
			Minor deficiencies: −1 to −5.5
			Major deficiencies: −6 to −10.5
			Massive deficiencies: −11 to −40
Rating 2	Individual criteria scores	Negative points assigned for each of the four criteria: odor, texture, color, impurities	Odor: 0 to −10
			Texture: 0 to −5
			Color: 0 to −5
			Impurities: 0 to −20
Rating 3	Binary classification of criteria	Each of the four criteria scored as 0 = acceptable, 1 = problematic	Odor2: 0 vs. 1
			Texture2: 0 vs. 1
			Color2: 0 vs. 1
			Impurities2: 0 vs. 1

**Table 4:** Number of microbial groups exceeding VDLUFA (Verband Deutscher Landwirtschaftlicher Untersuchungs- und Forschungsanstalten) guideline values per microbiological score level. Grades were assigned based on the highest number of exceedances. Multiple exceedances per sample were possible.

Grade	AMB FF	AMB Spoilage	AMB Strepto	Molds FF	Molds Spoilage	Molds Mucorales	Yeasts	Number of Hay
1	0	0	0	0	0	0	0	23
2	3	0	0	12	0	1	8	15
3	0	0	0	0	2	1	0	3
4	0	1	1	0	8	1	0	9

Abbreviations: AMB, aerobic mesophilic bacteria; FF, Field Flora; Spoilage, Spoilage-indicating; Strepto, Streptomycetes.

**Table 5:** Linear regression results for the outcome square root of particulate matter (PM) < 4 um in the Hay-Shaker and the binary predictors odor, texture, color and impurities as evaluated in the sensory examination (Rating 3). Intercept and coefficients with their corresponding *p*-values for the initial model are shown on the left, and for the final model after stepwise selection on the right.

	Initial Model	*p*-Value	Final Model	*p*-Value
Intercept	1.93	<2 × 10^−16^	2.06	<2 × 10^−16^
Odor2	0.85	4.28 × 10^−6^	0.90	9.58 × 10^−7^
Texture2	0.15	0.39		
Color2	0.11	0.54		
Impurities2	0.28	0.21		

**Table 6:** Final model: odds ratio for impurity score (Rating 2) and Brant test result.

Final Model	Value	SE	t	*p*	OR	2.5%	97.5%	Brant
Impurities	−0.615	0.325	−1.891	0.059	0.541	0.229	0.900	1.000

Abbreviations: SE, standard error; t, t-statistic; *p*, *p*-value; OR, Odds ratio; 2.5%/97.5%, lower and upper bounds of the 95% confidence interval; brant, *p*-value from the Brant test assessing the proportional odds assumption.

## Data Availability

The original data presented in the study are openly available in Zenodo at [10.5281/zenodo.16412564].

## Supplementary Materials

The following supporting information can be downloaded at: https://www.mdpi.com/article/10.3390/ani15182688/s1, Table S1: Summary of linear regression models for PM1 fraction based on sensory examination ratings; Table S2: Summary of linear regression models for PM2.5 fraction based on sensory examination ratings; Table S3: Summary of linear regression models for PM4 fraction based on sensory examination ratings; Table S4: Summary of linear regression models for PM10 fraction based on sensory examination ratings; Table S5: Summary of linear regression models for PMT fraction based on sensory examination ratings; Table S6: Summary of linear regression models for microbiological scoring based on sensory examination ratings.

## Abbreviations

The following abbreviations are used in this manuscript:

PM	Particulate Matter
VDLUFA	Verband Deutscher Landwirtschaftlicher Untersuchungs- und Forschungsanstalten
CFU	colony-forming units
BIC	Bayesian Information Criterion
AMB	Aerobic mesophilic bacteria
